# Whole genome sequencing analysis of antibiotic resistant genes of *Shigella* species: A systematic review and meta-analysis

**DOI:** 10.1371/journal.pone.0334701

**Published:** 2025-10-28

**Authors:** Basha Ayele, Getenet Beyene, Zeleke Mekonnen, Ahmed Esmael, Abaysew Ayele, Dawit Hailu Alemayehu, Getachew Tesfaye Beyene

**Affiliations:** 1 Department of Medical Laboratory Science, College of Health Science and Medicine, Dilla University, Dilla, Ethiopia; 2 School of Medical Laboratory Sciences, Institution of Health Sciences, Jimma University, Jimma, Ethiopia; 3 Armauer Hansen Research Institute, Addis Ababa, Ethiopia; Ahvaz Jondishapour University of Medical Sciences Faculty of Medicine, IRAN, ISLAMIC REPUBLIC OF

## Abstract

**Background:**

In developing nations, *Shigella* species are the leading cause of epidemic dysentery, especially among children under five. Antibiotic resistance has spread quickly among *Shigella* species as a result of inappropriate antibiotic use, inadequacies of diagnostic facilities, unhygienic conditions, and insufficient healthcare practices. This review aimed to describe AMR genes of *Shigella* species analyzed globally via whole genome sequencing (WGS).

**Methods:**

Relevant papers were found via a literature search using the databases of Google Scholar, Web of Science, PubMed, and Scopus. Full-text primary studies published in English, WGS, *Shigella* serogroup, and AMR gene statistics had to be included in the articles. The comprehensive meta-analysis software was used for data analysis. The Der Simonian–Laird random effect model was utilized and statistical heterogeneity between studies is measured by the I^2^ and Cochran’s Q test.

**Results:**

Of the studies, resistant genes of *S. flexneri* was more studied and characterized. The overall prevalence of antibiotics resistance genes was in the range of 1.7% to 46.9% with **gyrA* S83*L** was the most frequent isolated revealed this gene as predominant in the quinolones resistant gene of *S. sonnei.* It was followed by *mphA* (resistant to macrolides) for *S. flexneri,* and *sul2* (resistant to folate synthesis inhibitors) for *S. dysenteriae* and *S. boydii*. Pooled prevalence of AMR gene in *Shigella* species significantly varied among the studies (p = 0.001). There was no significant amount of heterogeneity in *S. bodyii* (Q (4)) =1.938. p = 0.747, I^2^ = 0%) however in *S. flexneri* (I^2^ = 63%) and *S. sonnei* (I^2^ = 84%) showed high heterogeneity within the studies.

**Conclusion:**

Generally, there was considerable variation in the pooled prevalence of the AMR gene in *Shigella* species among the studies, with *S. flexneri* and *S. sonnei* showing the highest levels of heterogeneity. The effectiveness of treatment is seriously threatened by *Shigella’s* resistance to antibiotics. Therefore, it is imperative that *Shigella* species resistance be continuously monitored globally.

## 1. Introduction

The most frequent cause of epidemic dysentery worldwide, particularly in children under the age of five, is shigellosis, which is caused by different *Shigella* species primarily found in developing nations [1]. Based on serological and biochemical traits, *Shigella*can be divided into four serogroups: S*. dysenteriae, S. flexneri, S. boydii*, and *S. sonnei* [2]. It is the main cause of infant diarrhea with high mortality rate in developing nations [3]. Inadequate hand washing following urination or diaper changes, as well as direct transmission from person to person through the fecal-oral pathway are the main route of acquiring the pathogens [4]. Compared to other causes of gastroenteritis, this microorganism is extremely contagious because only ten bacilli are required to cause an infection [5]. Typically, shigellosis is a severe invasive infection of the human colon and rectum that leads to severe inflammation and tissue necrosis.

The issue of shigellosis may have received less attention due to underreporting of cases and the existence of other illnesses deemed more serious [6]. The pattern of antimicrobial resistance (AMR) varies geographically and within a single region, and *Shigella* serogroups are evolving resistance to commonly used antimicrobial medications [2]. Multidrug resistance (MDR) to Shigella species is becoming more common, which poses a major risk, particularly in developing nations where there are issues with nutrition and health problems [7]. Antibiotic resistance has spread quickly among many bacterial classes as a result of inappropriate antibiotic use, inadequacies laboratory facilities, unhygienic conditions, and insufficient healthcare practices [8].

As resistance grows, antibiotics gradually lose their efficacy, allowing bacteria to adapt and thrive in their presence. Reduced efflux transport, target modification, restricted drug uptake, and enzyme-catalyzed inactivation are the main mechanisms that lead to the development of antibiotic resistance [9]. Antibiotics are transported from inside to the outside of bacteria by a broad class of protein pumps called efflux pumps. Additionally, through a series of DNA modifications or the synthesis of specialized enzymes that alter the antibiotic’s targets, bacteria can become resistant to a particular class of antibiotics [10]. Antibiotic absorption, however, can be restricted by certain proteins that have the ability to bind to either the antibiotics or their targets. Additionally, bacteria produce enzymes that recognize and break down the structural components of antibiotics, rendering them inactive [11]. Research has also shown that post-translational processes can contribute to the development of bacterial resistance [12]. These resistance mechanisms are classified as intrinsic or predicted resistance (present in all strains/bacteria) or acquired (first discovered) [13].

The phylogenetic analysis of strains and genes isolated domestically and their relationships to those found abroad is largely absent from conventional molecular methods [14]. Whole genome sequencing (WGS) is currently being used by researchers instead of more conventional methods, such as pulsed-field gel electrophoresis (PFGE), because of its higher resolution [15]. Due to insufficient facilities for precise detection and antibiotic resistance gene testing, *Shigella* species isolation is still difficult in the majority of public health laboratories [16]. Despite the high prevalence of shigellosis, there is a dearth of summary information on the genes of *Shigella* species that are responsible resistant to different antibiotics. Therefore, it is required to review WGS studies regarding the antibiotic-resistant genes in *Shigella* species.

## 2. Methods

### 2.1. Search strategy

Published papers were reviewed using information from a thorough literature search that included details on AMR genes and the *Shigella* species. The methodology for the literature search and review was guided by the predefined study protocol (S1 File). Using a full search approach and double-checking reference lists, relevant papers were found via a literature search using the databases of Google Scholar,Web of Science, PubMed, and Scopus. The following special index search phrases (medical subject headings, or MeSH) and Boolean operations were used to conduct a literature search: “*Shigella*” AND “WGS” OR “Epidemiology” AND “Drug Resistance Gene, Microbial” AND “Dysentery, Bacillary/ epidemiology” AND “title and abstract.” The published studies containing epidemiological and/or clinical data were the main focus of the article search. Endnote version 20 (Clarivate Analytics, Philadelphia, PA, USA) was used to manage all of the records. A search of the literature was done between July 10, 2023, and December 26, 2023. The study group was limited to humans, and the language was limited to English.

### 2.2. Eligibility criteria

**Fig 1:**
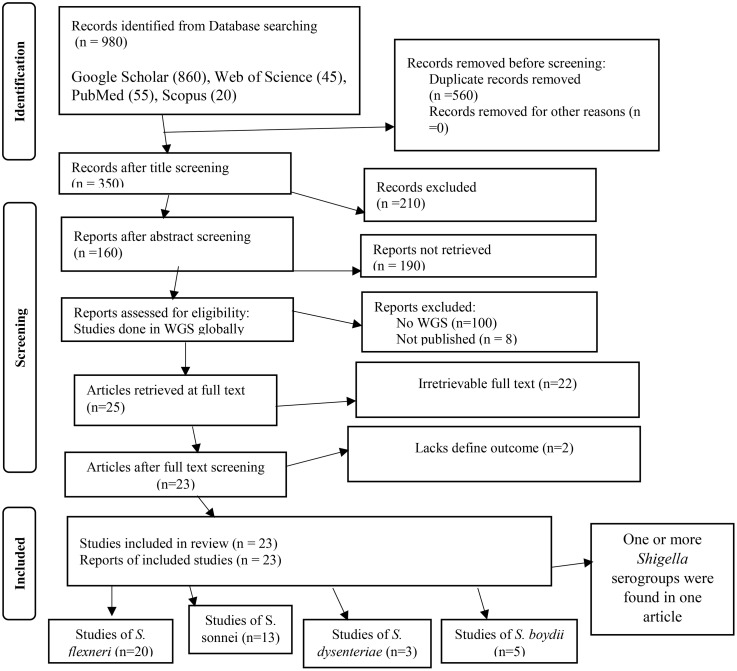
Chosen recording items for systematic reviews and meta-analysis flow chart for the selection of studies incorporated in the systematic review and meta-analysis.

Reviewed abstracts from the first search using the Preferred Reporting Items for Systematic reviews and Meta-Analyses (PRISMA) statement’s (S2 File) serogroups and outcome approach as a guide for defining inclusion and exclusion criteria. All WGS studies in *Shigella* serogroups were examined; however, the remaining studies were not provided because of the requirements for article inclusion. The *Shigella* serogroups and the AMR genes studies using the WGS method were the focus of the study’s outcome search. For the review to be included, full-text primary studies published in English, WGS, *Shigella* serogroup, and AMR gene statistics had to be included in the articles (Fig 1).

Exclusion criteria: Unpublished thesis and dissertations as well as papers without the required information were excluded. Since we are unable to evaluate the quality of each article in the absence of their full texts, studies that were not fully accessed after reading the titles and abstracts were excluded. Following the completion of the searches, every record that was found was downloaded and kept in EndNote 20 (Thompson Reuters) in a single library.

### 2.3. Data extraction

The researcher (BA) extracted data using a pretested, standardized format created in Microsoft Excel (S3 File). First author, study design, region, publication year, sample size, population characteristics, prevalence of *Shigella* species, and resistance gene were all included in the data abstraction format. When not enough information was provided, the article as a whole was examined to decide whether or not it belonged there. The reviewer (BA) both manually and automatically removed duplicates from the EndNote library in order to select which studies to include in the narrative synthesis. The same reviewer then went through the remaining records, selecting them first by the abstract and then by the title. After that, the full texts of the shortlisted articles were obtained to assess their eligibility for final inclusion. Prior to being implemented, the extraction sheet format was tested in 5% of the randomly selected studies. On the basis of a full-text analysis, the article was included. When data was extracted, publications were thoroughly assessed due to variations in study design.

### 2.4. Quality assessment

The Joanna Briggs Institute (JBI) eight-point critical appraisal tools were used to evaluate quality. The established criteria include: a sample frame that is appropriate for the target population; study participants who are sampled appropriately; detailed descriptions of the study subjects and setting; data analysis that covers a sufficient portion of the identified sample; valid methods for identifying the condition; a standard and reliable method of measuring the condition for every participant; appropriate statistical analysis; and a sufficient response rate. According to the reviews’ goals, a score that was calculated using various parameters was given to each study. “No and not reported” received a score of 0 while “Yes” received a score of 1. The range of total scores was 0–8. Research with medium quality, which satisfied 50% of the quality assessment criterion, and high quality were incorporated into the analysis [17].

### 2.5. Statistical analysis

The comprehensive meta-analysis software was used for the data analysis. Figures, tables, funnel and forest plots were used to describe the original articles. The random effect model was utilized to calculate the *Shigella* species’ pooled prevalence and antimicrobial resistance gene due to the heterogeneity among the studies. A 95% confidence interval (CI) for the estimated pooled prevalence rate was provided. Based on the serogroups of *Shigella* (*S. dysenteriae, S. flexneri, S. boydii, and S. sonnei*), sub-group analysis was carried out. I statistic and the Cochran’s Q test were used to assess heterogeneity. The I^2^ gives an estimate of the proportion of effect estimate variability attributable to heterogeneity as opposed to chance differences or sampling error. Therefore, the Cochran’s Q test was used to confirm the presence of heterogeneity (p < 0.10 indicates statistically significant heterogeneity). The statistical heterogeneity between studies is measured by the I^2^ test. I^2^ values of ≤25%, 25 < I^2^ ≤ 50%, and >50%, respectively, are considered to indicate low, medium, and high heterogeneity [18].

## 3. Results

### 3.1. Antimicrobial resistance gene of *Shigella* species

**Fig 2:**
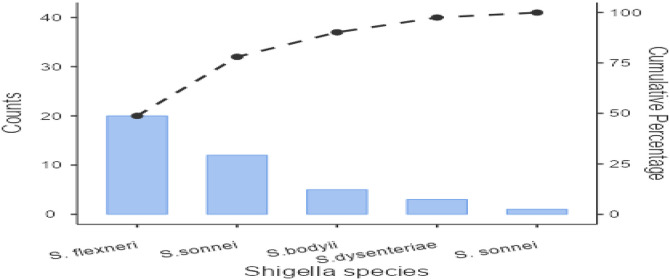
Pareto Chart for the prevalence of AMR *Shigella* species.

**Fig 3:**
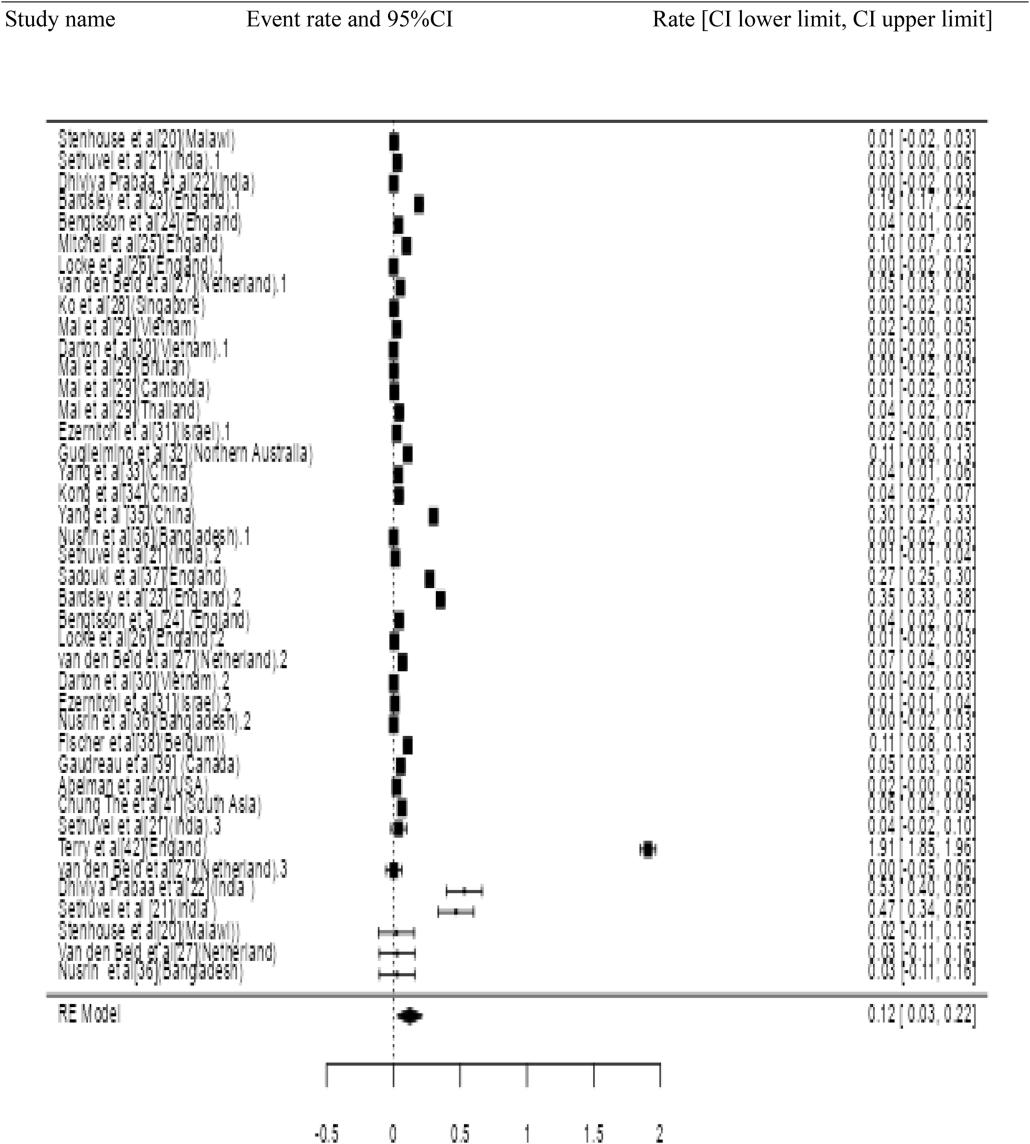
Forest plot for the prevalence of AMR genes of *Shigella* species.

**Fig 4:**
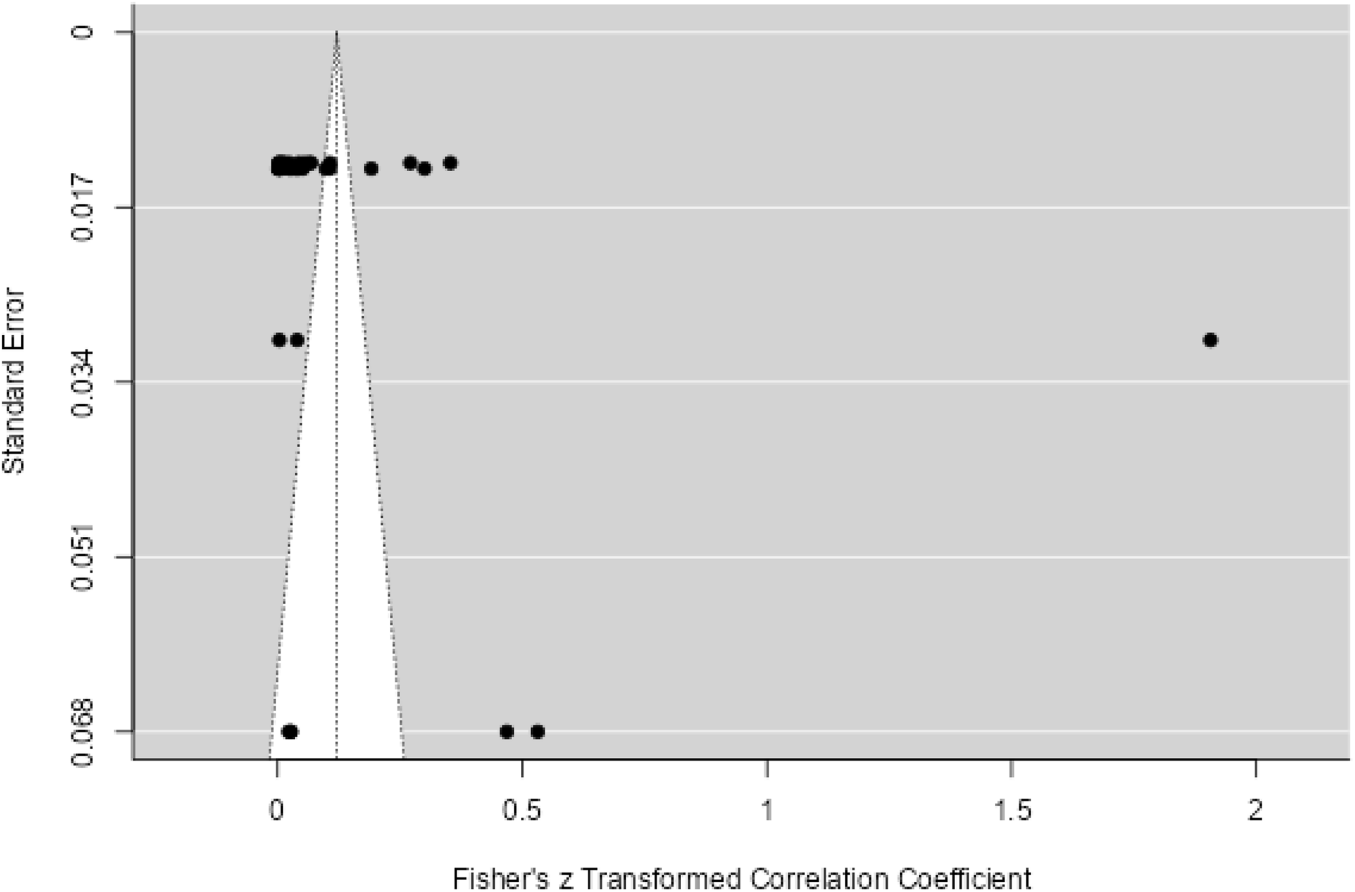
Funnel plot for the prevalence of AMR genes of *Shigella* species.

Twenty three studies with 4658 samples were included in our review of 280 titles and abstracts, including 20 studies of *S. flexneri* research, 13 studies of *S. sonnei*, 3 studies of *S. dysenteriae* and 5 studies of *S. boydii* (Fig 1). The reviewed studies included 39 sample sizes with the smallest and 2468 samples with the largest for *S. boydii* and *S. sonnei*, respectively (Tables 3 and 6). The study was included clinical samples from diarrheic patients with six studies were done on men who have sex with men (MSM). A number of resistant genes of *S. flexneri* was more studied and characterized than others (Table 2 and Fig 2). The overall prevalence of antibiotics resistance genes was in the range of 1.7% to 46.9% with **gyrA* S83L* was the most frequent isolated revealed this gene as predominant in the quinolones resistant gene of *S. sonnei*. I was followed by *mphA* (resistant to macrolides) for *S. flexneri,* and *sul2* (resistant to folate synthesis inhibitors) for *S. dysenteriae* and *S. boydii* (Tables 1–6). The analysis of 23 studies, according to the Der Simonian–Laird random-effects model, revealed that the pooled prevalence of antibiotics resistant gene was 12% (95% CI −0.07–0.17) (Figs 3 and 4). Pooled prevalence of AMR gene in *Shigella* species significantly varied among the studies (p = 0.001), with 9% in *S. flexneri*, 11% in *S. sonnei*, and 48% in *S. boydii*. The proportion of *S. dysenteriae* resistant genes was 33% however pooled prevalence was not estimated due to few study observations than the number of parameter estimated. According to the Q test there was no significant amount of heterogeneity in *S. bodyii* (Q(4))=1.9379. p = 0.7472, I^2^ = 0%) however in *S. flexneri* (I^2^ = 63%) and *S. sonnei* (I^2^ = 84%) showed high heterogeneity within the studies (–).

**Table 1:** Summary of 20 studies reporting the resistance genes with class of antimicrobials for *Shigella flexneri* in different regions.

Region	Author	publication year	study participants on sexuality	Specimen	Sample size for WGS(N) (1944 isolates)	Resistance to quinolones/fluoroquinolones	Resistance to folate synthesis inhibitors	Resistance to macrolides	Resistance to tetracyclines resistance	Resistance to phenicols	Resistance to aminoglycosides resistance	Resistance to β-lactams	Total detected resistance genes(%)	Quality score (8 points)
Malawi	Stenhouse et al [33]	2021	non specified	stool	5	1	11	0	5	3	7	2	29 (0.5)	4
India	Sethuvel et al [19]	2019	non specified	stool	23	45	40	0	20	0	43	17	169 (2.9)	6
India	Dhiviya Prabaa et al [32]	2017	non specified	stool	3	0	2	0	2	0	7	2	13 (0.2)	4
England	Bardsley et al [21]	2020	non specified	stool	891	312	0	780	0	0	0	0	1092 (18.9)	8
England	Bengtsson et al [22]	2021	MSM	stool	68	2	8	0	64	68	8	66	216 (3.7)	8
England	Mitchell et al [34]	2019	MSM	stool	391	0	0	566	0	0	0	0	566 (9.8)	8
England	Locke et al [23]	2021	MSM	stool	1	0	0	3	1	1	0	1	7 (0.1)	4
Netherland	van den Beld et al [24]	2023	MSM	stool	52	18	53	22	47	39	76	51	296 (5.1)	8
Singapore	Ko et al [35]	2022	non specified	stool	5	5	10	0	0	0	0	10	25 (0.4)	4
Vietnam	Mai et al [36]	2021	non specified	stool	25	1	43	2	23	21	29	23	142 (2.5)	6
Vietnam	Darton et al [25]	2018	non specified	stool	6	0	0	7	0	0	0	0	7 (0.1)	4
Bhutan	Mai et al [36]	2021	non specified	stool	6	0	11	0	5	0	5	0	21 (0.4)	4
Cambodia	Mai et al [36]	2021	non specified	stool	6	0	18	0	6	5	0	5	40 (0.7)	4
Thailand	Mai et al [36]	2021	non specified	stool	45	0	53	0	45	34	58	34	254 (4.4)	7
Israel	Ezernitchi et al [26]	2019	non specified	stool	19	5	32	24	12	7	33	31	144 (2.5)	5
Northern Australia	Guglielmino et al [37]	2020	non specified	stool	108	31	144	4	108	108	108	110	613 (10.6)	8
China	Yang et al [38]	2016	non specified	stool	24	76	26	0	0	0	51	55	208 (3.6)	6
China	Kong et al [39]	2023	non specified	stool	109	97	0	14	28	30	11	62	242 (4.2)	8
China	Yang et al [40]	2020	non specified	stool	152	495	262	25	422	0	280	199	1683 (29.1)	8
Bangladesh	Nusrin et al [27]	2022	non specified	stool	5	0	0	8	0	0	0	0	8 (0.1)	4
Total (%)						1089 (18.9)	743 (12.9)	1455 (25.2)	788 (13.6)	316 (5.5)	713 (12.3)	671 (11.6)	5775 (100)	

**Table 2:** Antimicrobial resistance genes reviewed in this paper with class of antimicrobials and expected to confer resistance to *Shigella flexneri* isolates.

Resistance to quinolones/fluoroquinolones (%)		Resistance to folate synthesis inhibitors (%)		Resistance to macrolides (%)		Resistance to tetracyclines (%)		Resistance to phenicols (%)		Resistance to aminoglycosides (%)		Resistance to β-lactams (%)	
*gyrA S83L*	330	*dfrA1*	405	*mphA*	756	*tetA*	176	*catA1*	315	*strA(aph(3’‘)-Ib)*	132	*blaTEM-1*	68
*gyrA D87G*	105	*dfrA3*	1	*mphE*	1	*tetB*	474	*floR*	1	*strB(aph(6)-Id)*	131	*blaTEM-1B*	37
*gyrA D87Y*	2	*dfrA7*	12	*msrE*	1	*tetD*	138			*aac(6′)-Ib-cr*	7	*blaTEM-141*	1
*gyrA H211T*	142	*dfrA12*	3	*ermB*	696					*aac(3)-IId*	10	*blaTEM-206*	1
*gyrA H211Y*	24	*dfrA14*	22	*mdfA*	1					*aac(3)-II*	1	*blaOXA-1*	474
*gyrA S87N*	18	*dfrA17*	13							*aac(3)-IIa*	16	*blaDHA-1*	8
*gyrA L4P*	2	*sul1*	57							*aadA1*	371	*blaCTX-1*	17
*gyrA A87A*	51	*sul2*	223							*aadA5*	18	*blaCTX-M-3*	2
*gyrA A87G*	6	*sul3*	7							*aadA16*	3	*blaCTX-M-9*	22
*gyrB Q776L*	2									*sat1*	24	*blaCTX-M-14*	13
*parC S80I*	321											*blaCTX-M-15*	11
*parC Q506L*	3											*blaCTX-M-27*	1
*parC R86C*	1											*blaCTX-M-55*	3
*QnrS*	12											*blaCTX-79*	1
*qnrS1*	36											*blaCTX-M-123*	1
*qnrA1*	1											*blaEC*	8
*qnrB*	27											*blaCMY-4*	1
*qnrB1*	1											*blaSHV-12*	2
*qnrB4*	3												
*qepA*	1												
*qepA2*	1												
1089(18.9)		743(12.9)		1455(25.2)		788(13.6)		316(5.5)		713(12.3)		671(11.6)	

**Table 3:** Summary of 13 studies reporting the resistance genes with class of antimicrobials for *Shigella sonnei* in different regions.

Region	Author	Publication year	Study participants on sexuality	Specimen	Sample size for WGS(N) (2468 isolates)	Resistance to quinolones/fluoroquinolones	Resistance to folate synthesis inhibitors	Resistance to macrolides	Resistance to tetracyclines	Resistance to phenicols	Resistance to aminoglycosides	Resistance to β-lactams	Total detected resistance genes (%)	Quality score (8 points)
India	Sethuvel et al [19]	2019	non specified	stool	15	30	28	0	1	0	25	5	89(1.4)	5
England	Sadouki et al [20]	2017	non specified	stool	341	108	659	55	0	9	713	123	1667(26.5)	8
England	Bardsley et al [21]	2020	non specified	stool	1140	1323	0	812	0	0	0	0	2135(33.9)	8
England	Bengtsson et al [22]	2021	MSM	stool	46	57	76	0	30	0	106	3	272(4.3)	8
England	Locke et al [23]	2021	MSM	stool	3	5	6	9	2	0	19	3	44(0.7)	4
Netherland	van den Beld et al [24]	2023	MSM	stool	55	76	151	33	42	0	135	18	430(6.8)	8
Vietnam	Darton et al [25]	2018	non specified	stool	16	0	0	16	0	0	0	0	16(0.3)	5
Israel	Ezernitchi et al [26]	2019	non specified	stool	12	0	14	24	0	0	16	12	66(1)	5
Bangladesh	Nusrin et al [27]	2022	non specified	stool	4	0	0	12	0	0	0	0	12(0.2)	4
Belgium	Fischer et al [28]	2022	MSM	stool	372	600	4	38	0	0	4	25	671(10.7)	8
Canada	Gaudreau et al [29]	2022	MSM	stool	31	31	62	93	31	0	93	31	341(5.4)	6
USA	Abelman et al [30]	2019	non specified	stool	22	19	48	3	21	2	48	9	150(2.4)	6
South Asia	Chung The et al [31]	2019	non specified	stool	411	402	0	0	0	0	0	0	402(6.4)	8
Total (%)						2651 (42.1)	1033 (16.4)	1095 (17.4)	127 (2)	11 (0.2)	1149 (18.3)	229 (3.6)	6295 (100)	

**Table 4:** Antimicrobial resistance genes reviewed in this paper with class of antimicrobials and expected to confer resistance to *Shigella sonnei* isolates.

Resistance to quinolones/fluoroquinolones (%)		Resistance to folate synthesis inhibitors (%)		Resistance to macrolides (%)		Resistance to tetracyclines (%)		Resistance to phenicols (%)		Resistance to aminoglycosides (%)		Resistance to β-lactams (%)	
*gyrA S83L*	928	*dfrA1*	496	*mphA*	514	*tetA*	120	catA1	11	*strA(aph(3’‘)-Ib)*	413	*blaTEM-1*	68
*gyrA D87G*	618	*dfrA5*	5	*ermB*	531	*tetB*	7			*strB(aph(6)-Id)*	406	*blaTEM-1B*	32
*gyrA D87Y*	225	*dfrA14*	17	*ermE*	4					*aac(3)-IId*	6	*blaTEM-1C*	5
*parC S80I*	846	*dfrA17*	38	*mdfA*	46					*aac(3)-Iva*	1	*blaTEM-117*	4
*qnrS1*	16	*dfrAB*	2							*aadA1*	287	*blaOXA-1*	12
*qnrB19*	18	*sul1*	46							*aadA5*	33	*blaDHA-1*	1
		*sul2*	429							*sat2*	3	*blaCTX-M-3*	5
												*blaCTX-M-14*	2
												*blaCTX-M-15*	30
												*blaCTX-M-27*	70
2651 (42.1)		1033 (16.4)		1095 (17.4)		127 (2)		11 (0.2)		1149 (18.3)		229 (3.6)	

Study name Event rate and 95%CI Rate [CI lower limit, CI upper limit].

**Table 5:** Summary of 3 studies reporting the resistance genes with class of antimicrobials for *Shigella dysenteriae* in different regions.

Region	Author	Publication year	study participants on sexuality	Specimen	Sample size for WGS(N) (207 isolates)	Resistance to quinolones/ fluoroquinolones	Resistance to folate synthesis inhibitors	Resistance to tetracyclines	Resistance to phenicols	Resistance to aminoglycosides	Resistance to β-lactams	Total detected resistance genes (%)	Quality score (8 points)
India	Sethuvel et al [19]	2019	non specified	stool	5	15	15	5	0	6	4	45(4)	4
England	Terry et al [41]	2018	non specified	stool	201	52	344	141	66	334	148	1085(95.7)	8
Netherland	van den Beld et al [24]	2023	MSM	stool	1	0	0	1	1	1	1	4(0.4)	4
Total (%)						67 (6)	359 (31.7)	147 (13)	67 (5.9)	341 (30)	153 (13.5)	1134 (100)	

**Table 6:** Summary of 5 studies reporting the resistance genes with class of antimicrobials for *Shigella boydii* in different regions.

Region	Author	Publication ear	Study participants on sexuality	Specimen	Sample size for WGS(N)	Resistance to quinolones/ fluoroquinolones	Resistance to folate synthesis inhibitors	Resistance to macrolides	Resistance to tetracyclines	Resistance to aminoglycosides	Resistance to β-lactams	Total detected resistance genes (%)	quality score (8 points)
India	Dhiviya Prabaa et al [32]	2017	non specified	stool	17	37	24	0	12	26	9	108 (48.6)	5
India	Sethuvel et al [19]	2019	non specified	stool	17	18	29	0	8	29	13	97 (43.7)	5
Malawi	Stenhouse et al [33]	2021	non specified	stool	1	0	1	0	1	2	1	5 (2.3)	4
Netherland	Van den Beld et al [24]	2023	MSM	stool	1	0	2	0	1	3	0	6 (2.7)	4
Bangladesh	Nusrin et al [27]	2022	non specified	stool	3	0	0	7	0	0	0	6 (2.7)	4
Total (%)						55 (24.8)	56 (25)	6 (2.7)	22 (9.9)	60 (27)	23 (10.4)	222 (100)	

**Fig 5:**
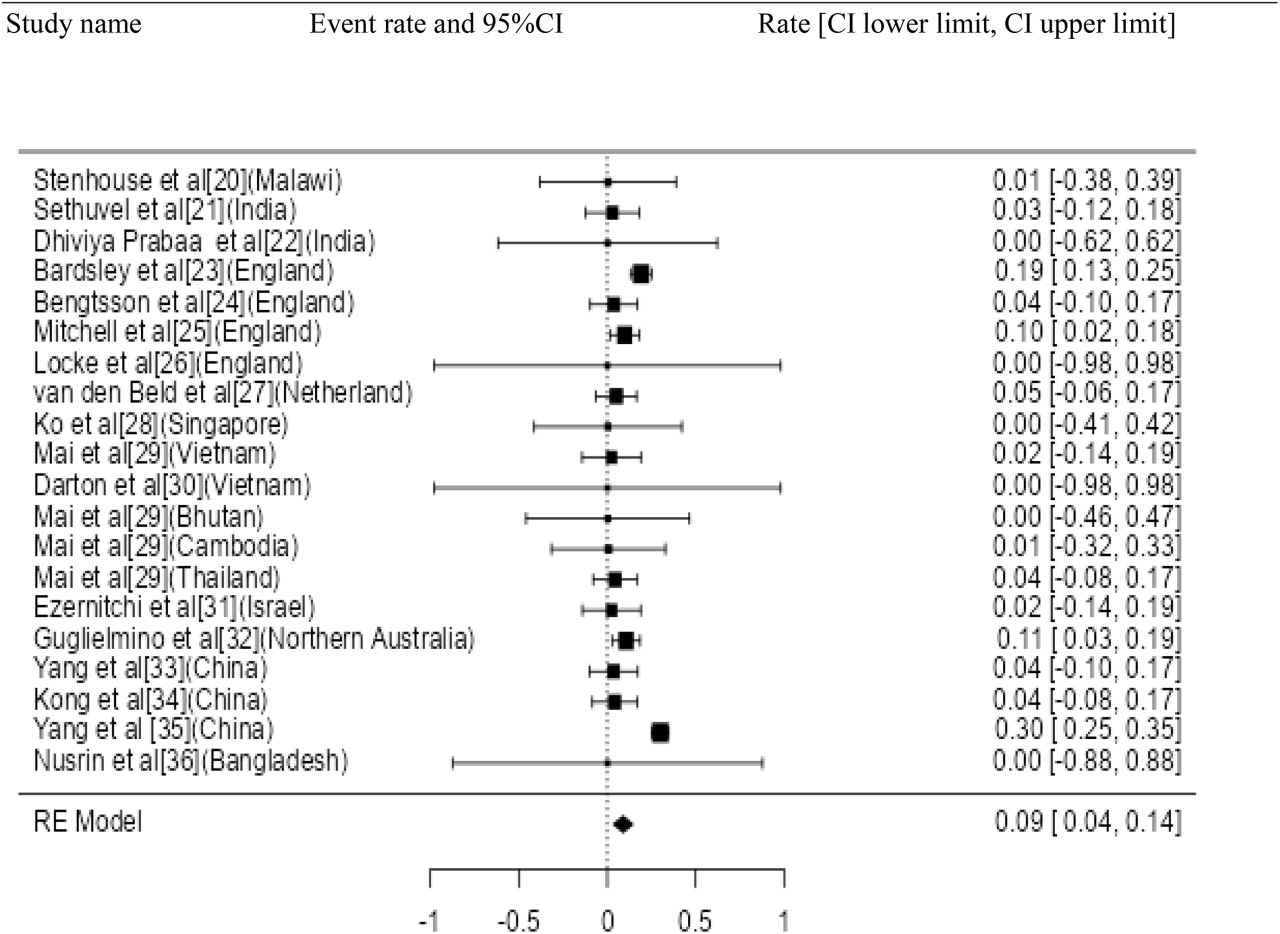
Forest plot for the prevalence of AMR genes of *S. flexneri.*

**Fig 6:**
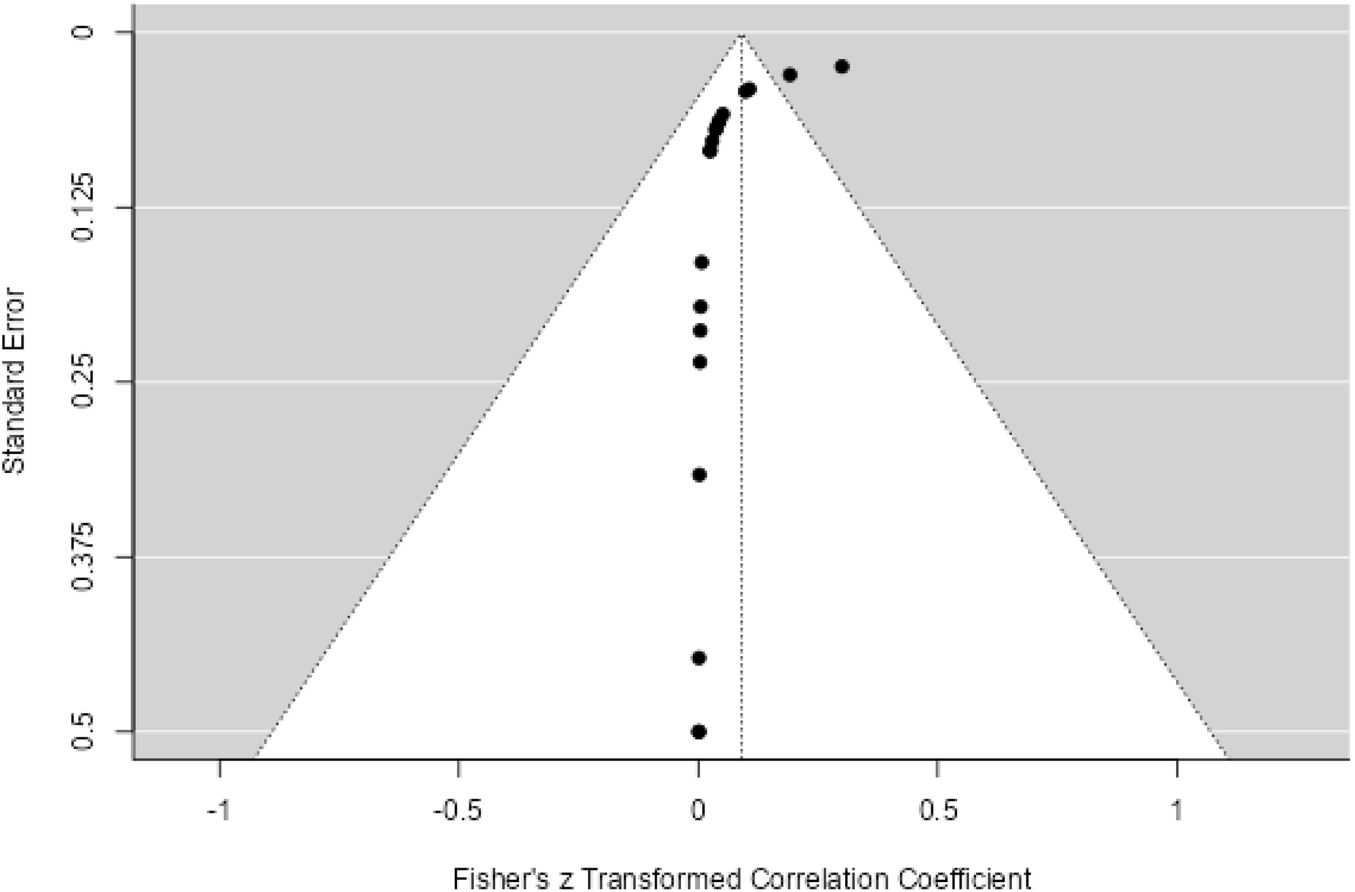
Funnel plot for the prevalence of AMR genes of *S. flexneri.*

**Fig 7:**
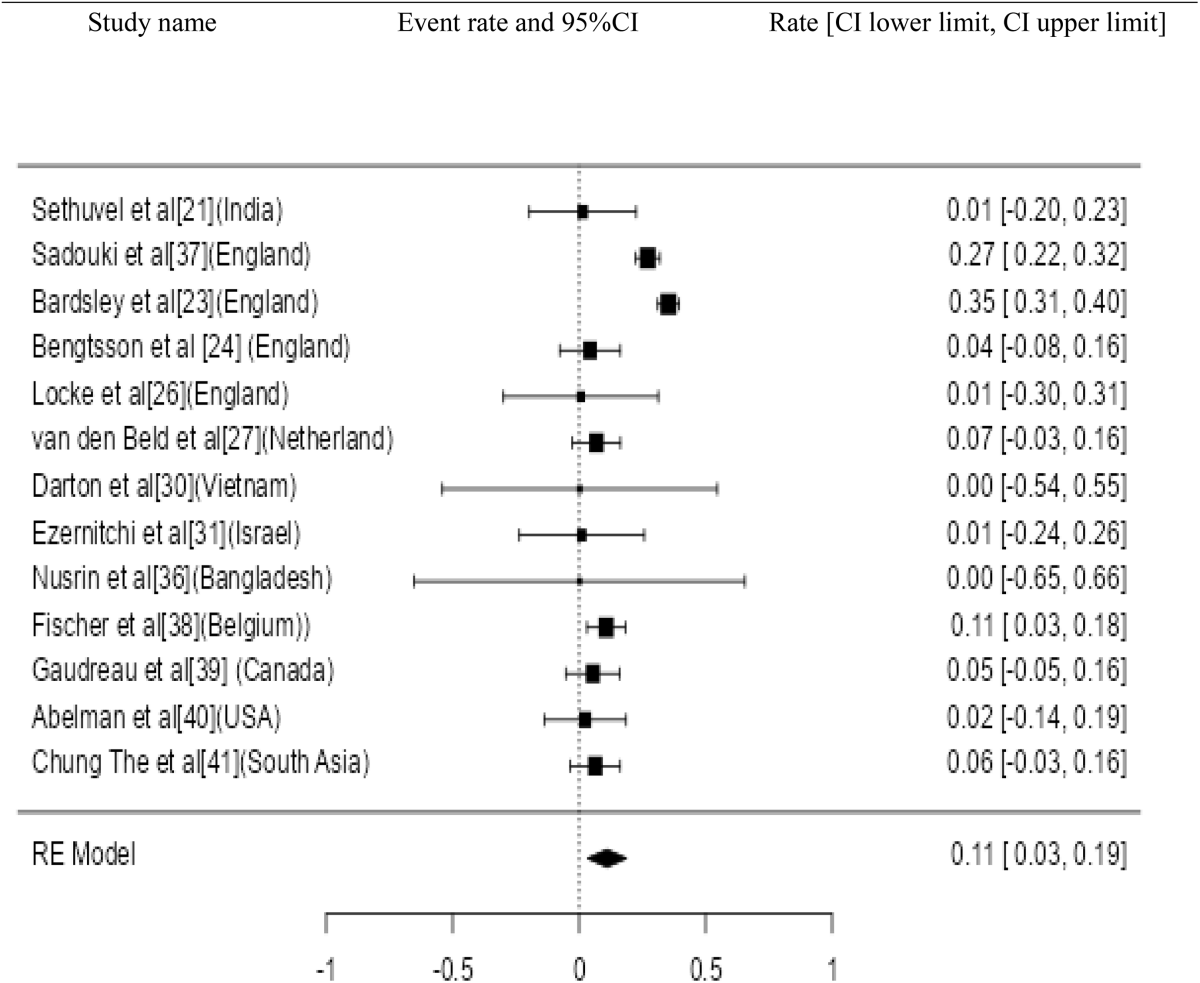
Forest plot for the prevalence of AMR genes of *S. sonnei.*

**Fig 8:**
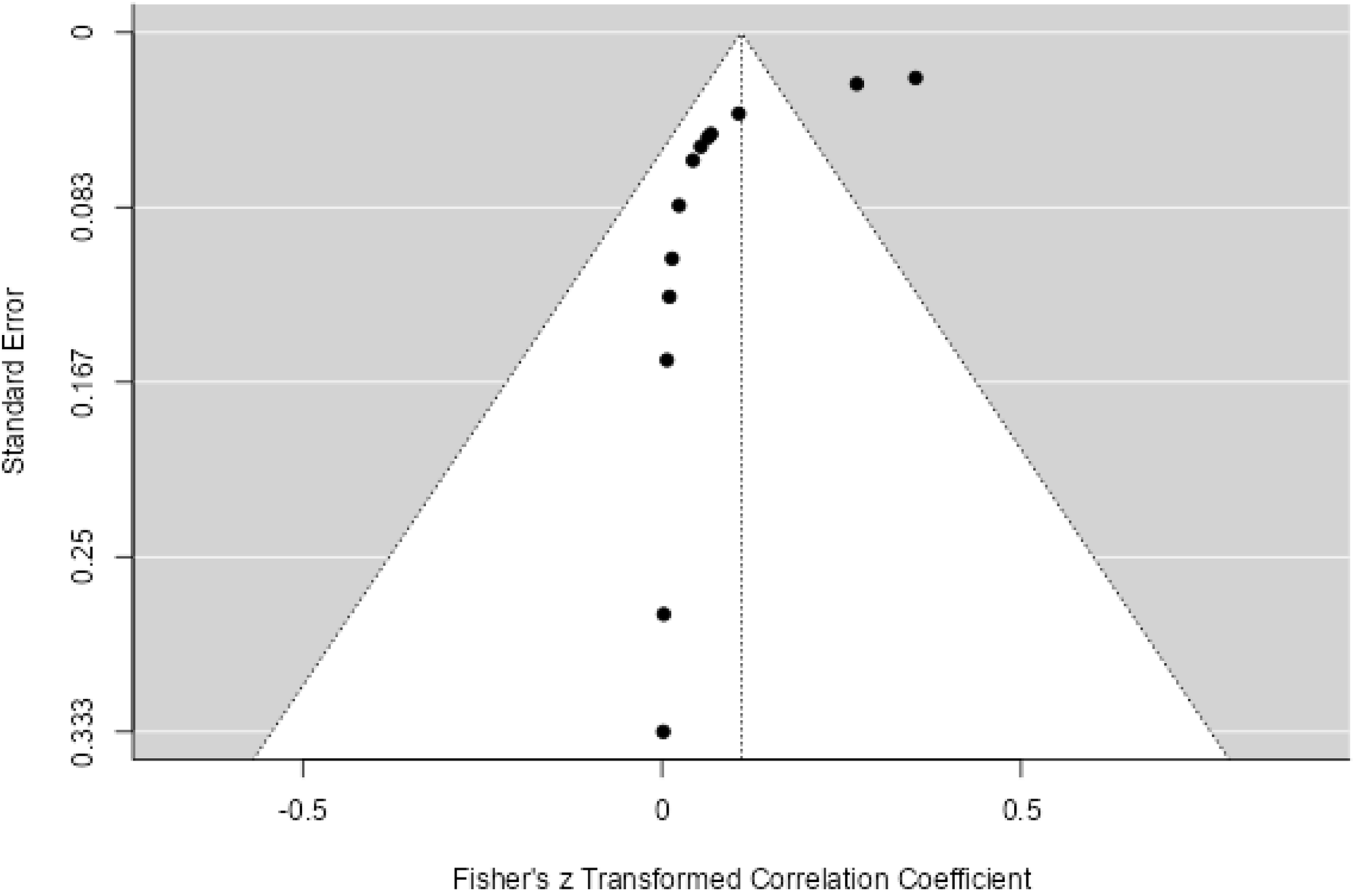
Funnel plot for the prevalence of AMR genes of *S. sonnei.*

**Fig 9:**
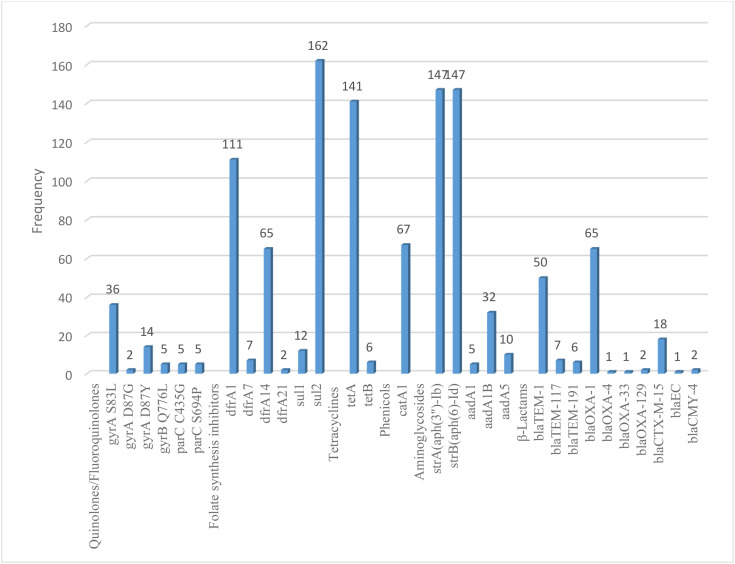
AMR genes of *S. dysenteriae.*

**Fig 10:**
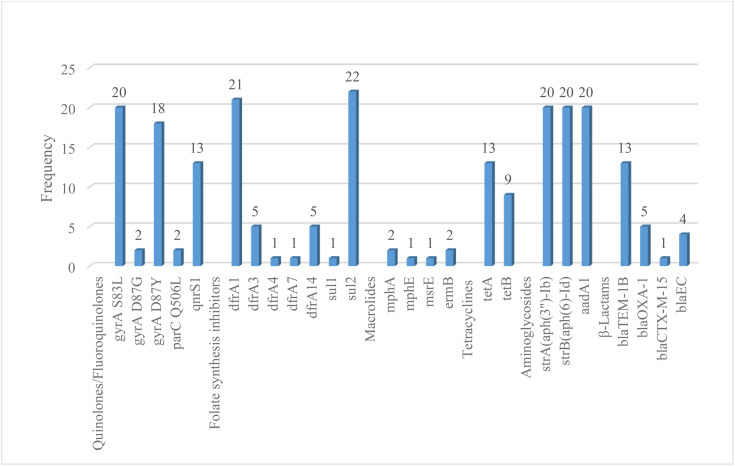
AMR genes of *S. boydii.*

**Fig 11:**
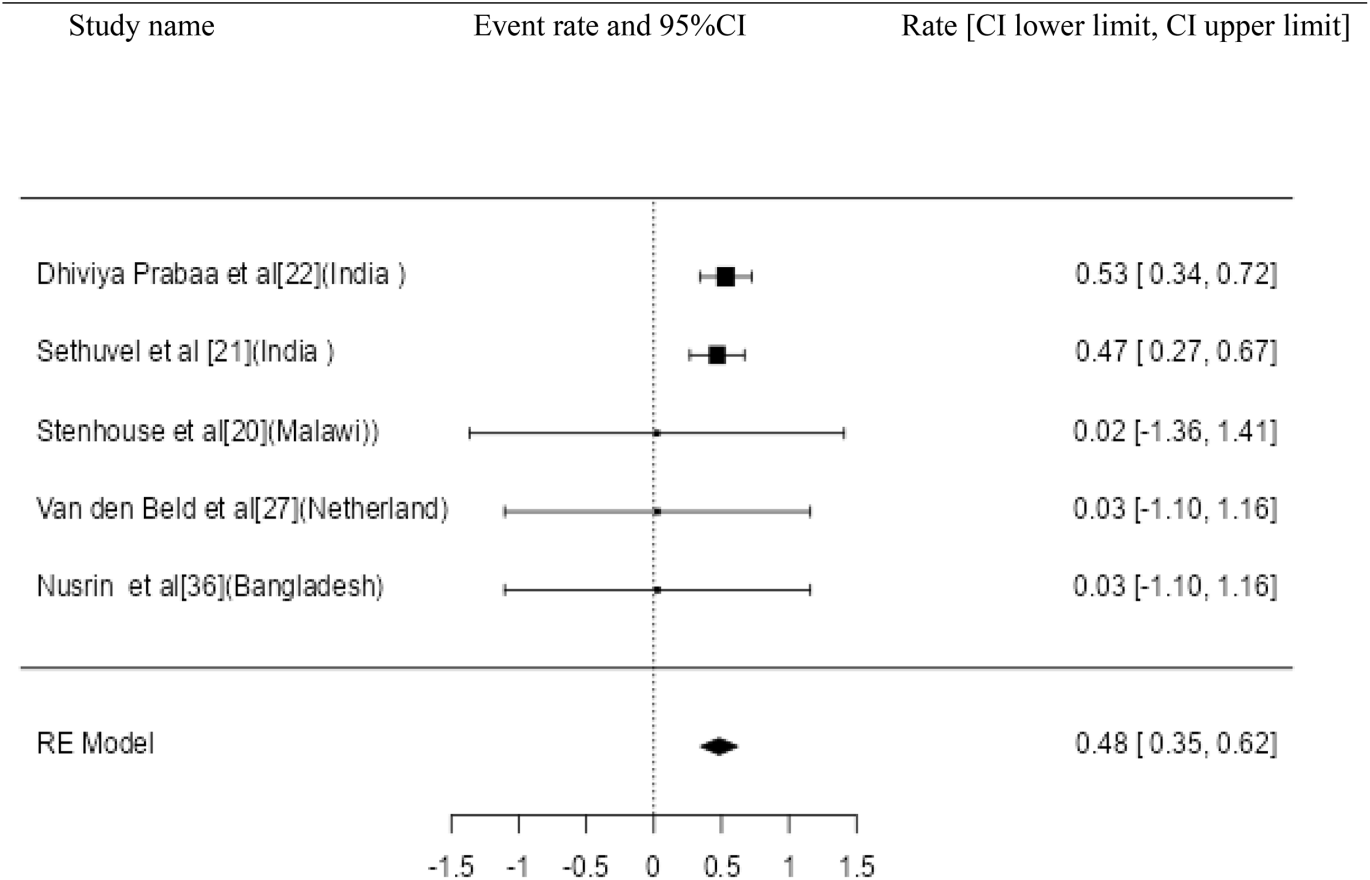
Forest plot for the prevalence of AMR genes of *S. boydii.*

**Fig 12:**
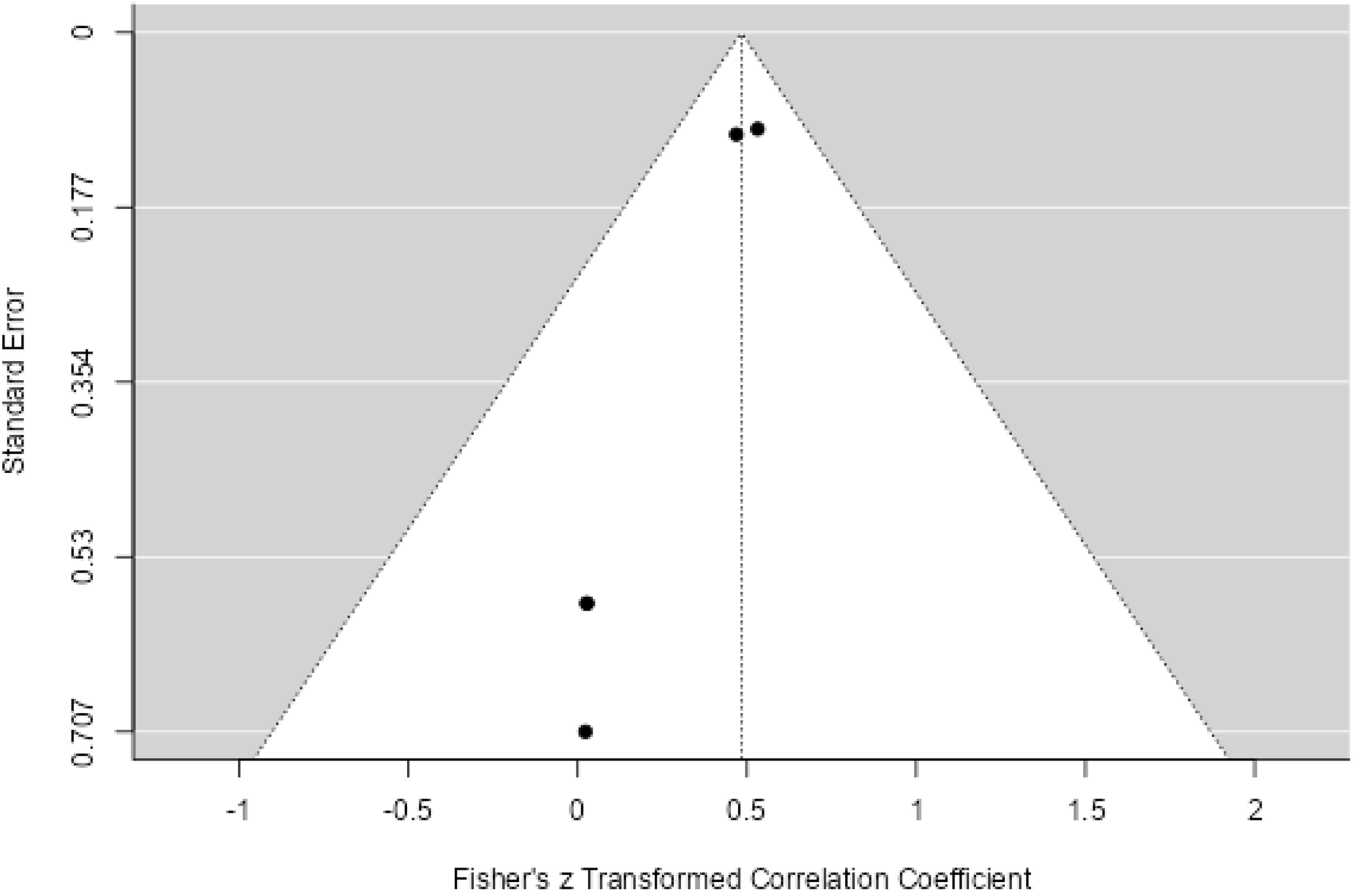
Funnel plot for the prevalence of AMR genes of *S. boydii.*

#### 3.1.1. Resistance to quinolones.

Three hundred thirty/5775 (5.7%), 928/6295(14.4%), 36/1134(3.2%), 20/222(9%) isolates had a gene mutation in *gyrA* leading to a *S83L* in *S. flexneri, S. sonnei, S. dysenteriae* and *S. boydii*, respectively. Additionally, 5.6% in *S. flexneri* and 13.4% *S. sonnei* in *parC* to *S80I* substitution but not detected from *S. dysenteriae* and *S. boydii*. Furthermore, 36(0.6%), 16(0.3%), zero and 13 (5.9%) isolates had the plasmid-mediated quinolone resistance determinant *qnrS1* in *S. flexneri, S. sonnei, S. dysenteriae* and *S. boydii*, respectively (Tables 1–4).

#### 3.1.2. Resistance to folate synthesis inhibitors.

Two hundred eight seven/5775 (5%) carried genes in *S. flexneri* conferring resistance to sulphonamides. Of these, 223(3.9%) had *sul2* (Table 1). About 6.8%, 14.5% and 9.9% were *sul2* for *S. sonnei, S. dysenteriae* and *S. boydii*, respectively. Four hundred fifty six/5775 (7.9%), 558/6295(8.9%), 185/1134(16.3%) and 33/222(14.9%) gene isolates carried conferring resistance to trimethoprim had 7%, 7.9%, 9.8% and 9.5% *dfrA1* in *S. flexneri, S. sonnei, S. dysenteriae* and *S. boydii,* respectively.

#### 3.1.3. Resistance to macrolides.

Of the isolated genes *mphA* and *ermB* genes potentially linked to conferring resistance to the macrolides. One thousand four hundred fifty five/5775 (25.2%) genes had 756(13.1%) *mphA* and 696(12.1%) *ermB* in *S. flexneri*, 1095(17.4%) genes had 514(8.2%) *mphA* and 531(8.4%) *ermB* in *S. sonnei*, 6(2.7%) genes had 2(0.9%) *mphA* and 2(0.9%) *ermB* in *S. boydii* but no gene detected in *S. dysenteriae* (Tables 1–4).

#### 3.1.4. Resistance to tetracyclines.

One thousand eighty four/13426 (8.1%) genes were confirmed resistance to tetracyclines. High number of *tetB*(8.2%) genes were detected in *S. flexneri* whereas 1.9%, 12.4% and 5.9% *tetA* genes were isolated in *S. sonnei, S. dysenteriae* and *S. boydii*, respectively (Tables 1–4).

#### 3.1.5. Resistance to phenicols.

Three hundred nighty four/13426 (2.9%) isolated genes conferring resistance to chloramphenicol. Of these, 315/5775(5.5%) were *catA1* and one *floR* only was found in *S. flexneri*. Eleven (0.2%) and 67(5.9%) genes were catA1 in *S. sonnei and S. dysenteriae*, respectively (Tables 1–4).

#### 3.1.6. Resistance to aminoglycosides.

The gene *strA, strB* and *aadA1* isolates resistance to streptomycin more confirmed than other genes resistance to aminoglycosides (Tables 1–4). Three hundred seventy one/5775 (6.4%) isolates had *aadA1* in *S. flexneri*. Four hundred thirteen/6295(6.6%) had *strA* and 6.4% had *strB* in *S. sonnei,* 12.9% had *strA-strB* in *S. dysenteriae* and 9% had *strA-strB* and aadA1 in *S. boydii*. Other low number of combinations of genes also confirmed resistance to a broad range of aminoglycosides, including streptomycin, gentamicin and tobramycin.

#### 3.1.7. Resistance to β-lactams.

Nine hundred seventy one/13426 (7.2%) gene isolates carried conferring resistance to β-lactams (Table 1–4). The penicillinases *blaOXA-1* (8.2%) in *S. flexneri, blaOXA-1* (5.7%) and *blaTEM-1*(4.4%) in *S. dysenteriae, blaTEM-1* (1%) in *S. sonnei*, *blaTEM-1B* (5.9%) in *S. boydii* and the extended-spectrum beta-lactamase (ESBLs) *blaCTX-M-27* (1.1%) in *S. sonnei* were frequently detected (Tables 1–4).

## 4. Discussion

Many studies have been conducted worldwide, even though they have concentrated on the phenotypic characteristics of *Shigella* species. This review study described the AMR genes of *Shigella* species. In developing nations with poorer standards of hygiene, Shigellosis can be regarded as a significant pathogen [42]. This review used 23 studies to determine the pooled prevalence of AMR genes of *Shigella* species in various regions. The review’s findings indicate that 12% of the population had a pooled prevalence of an antibiotic resistance gene. The studies’ pooled prevalence of the AMR gene varied significantly, and it has also been linked to MSM sexual transmission. The geographical origin of the isolate and/or variations in the routes of transmission may be connected to the variation in AMR that has been observed [43]. In Asia and Africa, shigellosis is endemic, primarily affecting children under the age of five [44]. In developed regions such as Europe, the Americas, and Australia, *Shigella* infections are more common in HIV-positive people, the homeless, and travelers. Moreover, a noteworthy proportion of *Shigella* infections are contracted via intercourse. They have greater MDR rates than *Shigella* isolates with different modes of transmission [45]. In this review, there were variations in drug resistance between the *Shigella* serogroups. The range of antibiotic-resistant gene prevalence was 1.7% to 46.9%. The most common isolate, **gyrA* S83L*, identified this gene as the predominant resistance gene to quinolones of *S. sonnei*. It was followed by *mphA* (resistant to macrolides) for *S. flexneri,* and *sul2* (resistant to folate synthesis inhibitors) for *S. dysenteriae* and *S. boydii.* This could be the third-generation cephalosporin resistance that emerged in 2014 and spread among MSM in Europe and America, along with resistance to azithromycin and ciprofloxacin [46]. In this review, *S. sonnei* exhibited higher rates of gene resistance than other serogroups. Compared to *S. flexneri, S. sonnei* may have a higher rate of drug resistance gene element transmission, especially for antibiotics like quinolones/fluoroquinolones and folate synthesis inhibitors [47,48]. In *S. flexneri* and *S. sonnei*, the pooled prevalence of the AMR gene varied significantly across the studies with a notable degree of heterogeneity, but not in *S. boydii*.

When *Shigella* species develop resistance to first and second-line antibiotics used in clinical settings, it is extremely concerning. There have been reports of *Shigella* species becoming resistant to ciprofloxacin and ceftriaxone [49]. Two distinct mutations in the *gyrA* gene and one single mutation in the *parC* gene are the main causes of ciprofloxacin resistance in bacteria. Furthermore, it has been found that the emergence of plasmid-mediated quinolone resistance and efflux pumps facilitates the development of resistance levels to quinolones and fluoroquinolones [50]. The *qnr* genes have been classified into five families: q*nrA1-7, qnrS1-4, qnrB1-31, qnrC*, and *qnrD.* Each family has a different number of alleles. It is typical to recognize *qnrA, qnrB*, and q*nrS* among these [45]. In this review, the most frequently observed *qnr* genes were *qnrS1* genes, followed by *qnrB* genes.

The gene that conferred resistance to sulphonamides in the current review was *sul2*, and the gene isolates that conferred resistance to trimethoprim had *dfrA1*. These genes are more acquired or co-occurring with *blaTEM* variants, *tet(A*) or *tet(B), aph(3“)-Ib (strA)* and/or *aph(6)-Id (strB),* or with *sul2* and *dfrA1* genes [51]. In this review, *tetA* genes were isolated from *S. sonnei, S. dysenteriae*, and *S. boydii*, while a high number of *tetB* genes were found in *S. flexneri*. According to a study by Mandomando et al., *S. flexneri* is associated with the presence of the *tetB* and *dfrA1* genes, whereas S*. sonnei* is primarily associated with the presence of the *tetA* and *dfrA1* genes [52]. Furthermore, compared to other genes resistant to aminoglycosides, this review demonstrated that isolates with the streptomycin resistance genes *strA, strB,* and *aadA1* were more confirmed. The majority of the *Shigella* isolates carry *strA/B, aadA1, tetA/B, catA1* and other genes that confer resistance to aminoglycosides, tetracycline and chloramphenicol [19].

The main mechanism of resistance for *Shigella* species to azithromycin is believed to be the presence of genes resistant to macrolides. Different bacteria have different mechanisms for resisting azithromycin. The *mphA* and *ermB* genes were reviewed in this study as possibly contributing to the macrolide resistance. The genetic structures *IS26-mph(A)-mrx(A)-mph(R)(A)-IS6100* and *mph(E)-msr(E)-IS482-IS6* that carry macrolide-resistant genes are found in *Shigella* [27]. Because of the increasing prevalence of resistance to ciprofloxacin and ceftriaxone, azithromycin is regarded as a last-resort, Food and Drug Administration (FDA)-approved antibiotic agent for the treatment of systemic infections, especially those caused by *Shigella* species [53].

In the current review, 7.2% of gene isolates had resistance to β-lactam antibiotics. The penicillinases *blaOXA-1* in *S. flexneri*, *blaOXA-1* and *blaTEM-1* in *S. dysenteriae, blaTEM-1* in *S. sonnei*, *blaTEM-1B* in *S. boydii* and the ESBLs *blaCTX-M-27* in *S. sonnei* were frequently detected*.* Plasmids carrying multiple AMR genes are commonly identified in *Shigella* species, and these include *IncFII, IncI1, IncI2*, and *IncB/O/K/Z* plasmids. These plasmids are capable of containing various AMR gene types [54]. *Shigella* species’ resistance to ceftriaxone is partially attributed to genes encoding ESBL, such as *blaTEM, blaSHV, blaCMY, blaCTX-M*, and *blaOXA* [49]*.*

Our study has some limitations. The information derived from our 23 WGS studies might not be universally representative of AMR genes. Our study’s findings were further constrained by insufficient data on AMR genes and the overall number of cases that were reported. A dearth of published data, particularly from Africa where *Shigella* was common, further restricted our study. As a result, the estimated AMR genes prevalence might not apply to other areas where studies assessing WGS *Shigella* species were lacking. On the other hand, *Shigella* AMR may be on the rise in most areas.

## 5. Conclusion

In summary, there was considerable variation in the pooled prevalence of the AMR gene in *Shigella* species among the studies, with *S. flexneri* and *S. sonnei* showing the highest levels of heterogeneity. The effectiveness of treatment is seriously threatened by *Shigella’s* resistance to antibiotics. Since this bacteria are multifactorial pathogen, drug resistance has varied noticeably in different serogroups, isolation sources, or geographic locations. Therefore, it is imperative that enteric *Shigella* resistance be continuously monitored globally.

## Supporting information

S1 File A2 Text: Study protocol. (PDF)

S2 File A Text: PRISMA checklist. (PDF)

S3 File Data collection form. (XLSX)
