# Supplementary material for: Whole genome sequencing analysis of antibiotic resistant genes of Shigella species: A systematic review and meta-analysis
Source: PLoS One. 2025 Oct 28;20(10):e0334701. doi: 10.1371/journal.pone.0334701 (PMC12561957; doi:10.1371/journal.pone.0334701)
Supplement: S1 File — (PDF) [file pone.0334701.s001.pdf]

**A2 Text. Study protocol.**

**Whole genome sequencing (WGS) analysis of antibiotic-resistant genes of *Shigella* species: A systematic review and meta-analysis**

## Study Protocol

## OBJECTIVES

This study is to investigate the epidemiological variation and antimicrobial resistance (AMR) of *Shigella* species globally.

**Primary objective**

To determine epidemiological variation of *Shigella* species globally:

- Prevalence of *Shigella* species globally
- Distribution of *Shigella* species globally

**Secondary objective**

To determine AMR epidemiology *Shigella* species were included:

- Acquired AMR genes of *Shigella* species
- Chromosomal mutation genes of *Shigella* species

## METHODS

**Study selection**

Inclusion criteria:

- Study design: WGS molecular data associated to AMR of *Shigella* species
- Outcomes:
    - Prevalence of *Shigella* species
    - Variation of *Shigella* species
    - Prevalence of acquired AMR genes of *Shigella* species
    - Prevalence of chromosomal mutation genes of *Shigella* species

Exclusion criteria:

- No original data or data duplication
- No WGS studies

- No *Shigella* species
- No AMR resistance

**Data abstraction**

The following information will be collected where available:

- Name or title of reports/author/journal
- Year of publication
- Study setting (region)
- Study design
- Sample size
- Characteristics of study participants (Ward/department, sub-group, if any)
- Outcomes: *Shigella* infection prevalence, variation of epidemiology, prevalence of AMR genes, Sample type, and study participants

**Search strategy**

Literature search will involve Google Scholar, Web of Science, PubMed, and Scopus via searching the references of original articles. Search will be limited to literature with at least a title, abstract, and keywords in the English language. Search terms combination will include: "*Shigella*" AND "WGS" OR "Epidemiology" AND "Drug Resistance Gene, Microbial" AND "Dysentery, Bacillary/ epidemiology" AND "title and abstract.

**Analysis plan**

- The following data will be illustrated (if data is available):
  - Prevalence of *Shigella* species
  - Variation of *Shigella* species
  - Prevalence of acquired AMR genes of *Shigella* species
  - Prevalence of chromosomal mutation genes of *Shigella* species
- Quality assessment
  - The Joanna Briggs Institute (JBI) eight-point critical appraisal tools were used to evaluate quality.
  - The established criteria include:

- a sample frame that is appropriate for the target population
- study participants who are sampled appropriately
- detailed descriptions of the study subjects and setting
- data analysis that covers a sufficient portion of the identified sample
- valid methods for identifying the condition
- a standard and reliable method of measuring the condition for every participant
- appropriate statistical analysis
- and a sufficient response rate.

**Data management**

The initial data analysis will address only the objectives specified above. Preliminary findings from these analyses will be circulated among contributing investigators for their comments and suggestions about further analysis. Any additional analysis will be proposed to all investigators; even though related to the objectives above, we will not seek unanimous approval of any such additional analyses. However, objections to the new analysis will be addressed and resolved before proceeding. (In other words, if a collaborator does not respond, that is taken as implicit approval). If we think of analyses to address entirely different and novel objectives that are not considered or foreseen here, we will seek approval from ALL investigators before embarking on any such analyses.

All proposed publications will be reviewed and approved by all investigators before public presentation or submission for publication. The authorship will include all responsible investigators contributing data.
