# Supplementary material for: Whole genome sequencing analysis of antibiotic resistant genes of Shigella species: A systematic review and meta-analysis
Source: PLoS One. 2025 Oct 28;20(10):e0334701. doi: 10.1371/journal.pone.0334701 (PMC12561957; doi:10.1371/journal.pone.0334701)
Supplement: S2 File — (PDF) [file pone.0334701.s002.pdf]

A Text. PRISMA checklist

Section and Topic	Item #	Checklist item	Reported on page
**TITLE**			
Title	1	Identify the report as a systematic review.	Title page
**ABSTRACT**			
Abstract	2	See the PRISMA 2020 for Abstracts checklist.	Abstract
**INTRODUCTION**			
Rationale	3	Describe the rationale for the review in the context of existing knowledge.	Introduction, Paragraphs 2-3
Objectives	4	Provide an explicit statement of the objective(s) or question(s) the review addresses.	Introduction, paragraph 4
**METHODS**			
Eligibility criteria	5	Specify the inclusion and exclusion criteria for the review and how studies were grouped for the syntheses.	Methods, paragraphs 2
Information sources	6	Specify all databases, registers, websites, organisations, reference lists and other sources searched or consulted to identify studies. Specify the date when each source was last searched or consulted.	Methods, search strategy and data extraction
Search strategy	7	Present the full search strategies for all databases, registers and websites, including any filters and limits used.	Methods, search strategy & Figure1
Selection process	8	Specify the methods used to decide whether a study met the inclusion criteria of the review, including how many reviewers screened each record and each report retrieved, whether they worked independently, and if applicable, details of automation tools used in the process.	Methods, data extraction and eligibility criteria
Data collection process	9	Specify the methods used to collect data from reports, including how many reviewers collected data from each report, whether they worked independently, any processes for obtaining or confirming data from study investigators, and if applicable, details of automation tools used in the process.	Methods, data extraction
Data items	10a	List and define all outcomes for which data were sought. Specify whether all results that were compatible with each outcome domain in each study were sought (e.g. for all measures, time points, analyses), and if not, the methods used to decide which results to collect.	Methods, data quality
	10b	List and define all other variables for which data were sought (e.g. participant and intervention characteristics,	Methods, data quality

Section and Topic	Item #	Checklist item	Reported on page
		funding sources). Describe any assumptions made about any missing or unclear information.	
Study risk of bias assessment	11	Specify the methods used to assess risk of bias in the included studies, including details of the tool(s) used, how many reviewers assessed each study and whether they worked independently, and if applicable, details of automation tools used in the process.	Methods, data extraction
Effect measures	12	Specify for each outcome the effect measure(s) (e.g. risk ratio, mean difference) used in the synthesis or presentation of results.	Methods, data analysis
Synthesis methods	13a	Describe the processes used to decide which studies were eligible for each synthesis (e.g. tabulating the study intervention characteristics and comparing against the planned groups for each synthesis (item #5)).	Methods, data quality
	13b	Describe any methods required to prepare the data for presentation or synthesis, such as handling of missing summary statistics, or data conversions.	Methods, data analysis
	13c	Describe any methods used to tabulate or visually display results of individual studies and syntheses.	Methods, data analysis
	13d	Describe any methods used to synthesize results and provide a rationale for the choice(s). If meta-analysis was performed, describe the model(s), method(s) to identify the presence and extent of statistical heterogeneity, and software package(s) used.	Methods, data analysis
	13e	Describe any methods used to explore possible causes of heterogeneity among study results (e.g. subgroup analysis, meta-regression).	Methods, data analysis
	13f	Describe any sensitivity analyses conducted to assess robustness of the synthesized results.	Methods, data analysis
Reporting bias assessment	14	Describe any methods used to assess risk of bias due to missing results in a synthesis (arising from reporting biases).	Methods, data analysis & data quality
Certainty assessment	15	Describe any methods used to assess certainty (or confidence) in the body of evidence for an outcome.	Methods, data analysis & data quality
**RESULTS**			
Study selection	16a	Describe the results of the search and selection process, from the number of records identified in the search to the number of studies included in the review, ideally using a	Results, paragraph 1; Fig 1-3

Section and Topic	Item #	Checklist item	Reported on page
		flow diagram.	
	16b	Cite studies that might appear to meet the inclusion criteria, but which were excluded, and explain why they were excluded.	Results, paragraph 1
Study characteristics	17	Cite each included study and present its characteristics.	Results, paragraphs 1, 2, 3
Risk of bias in studies	18	Present assessments of risk of bias for each included study.	Results, paragraph 1
Results of individual studies	19	For all outcomes, present, for each study: (a) summary statistics for each group (where appropriate) and (b) an effect estimate and its precision (e.g. confidence/credible interval), ideally using structured tables or plots.	Results, Fig 3-12
Results of syntheses	20a	For each synthesis, briefly summarise the characteristics and risk of bias among contributing studies.	Results, table1-6
	20b	Present results of all statistical syntheses conducted. If meta-analysis was done, present for each the summary estimate and its precision (e.g. confidence/credible interval) and measures of statistical heterogeneity. If comparing groups, describe the direction of the effect.	Results, Fig 3-12
	20c	Present results of all investigations of possible causes of heterogeneity among study results.	Results, Figure 5-8, 11,12
	20d	Present results of all sensitivity analyses conducted to assess the robustness of the synthesized results.	Results, table1,3,5,6
Reporting biases	21	Present assessments of risk of bias due to missing results (arising from reporting biases) for each synthesis assessed.	Results, table 5
Certainty of evidence	22	Present assessments of certainty (or confidence) in the body of evidence for each outcome assessed.	Results, table1,3,5,6
**DISCUSSION**			
Discussion	23a	Provide a general interpretation of the results in the context of other evidence.	Discussion, paragraph 1
	23b	Discuss any limitations of the evidence included in the review.	Discussion, paragraphs 6
	23c	Discuss any limitations of the review processes used.	Discussion, paragraph 6
	23d	Discuss implications of the results for practice, policy, and future research.	Discussion, Conclusion

Section and Topic	Item #	Checklist item	Reported on page
**OTHER INFORMATION**			
Registration and protocol	24a	Provide registration information for the review, including register name and registration number, or state that the review was not registered.	PROSPERO
	24b	Indicate where the review protocol can be accessed, or state that a protocol was not prepared.	A2 text; study protocol
	24c	Describe and explain any amendments to information provided at registration or in the protocol.	No amendment
Support	25	Describe sources of financial or non-financial support for the review, and the role of the funders or sponsors in the review.	Page 30 & Submission process
Competing interests	26	Declare any competing interests of review authors.	page 30 & Submission process
Availability of data, code and other materials	27	Report which of the following are publicly available and where they can be found: template data collection forms; data extracted from included studies; data used for all analyses; analytic code; any other materials used in the review.	A2 text & A3 Data collection form and Submission process

From: Page MJ, McKenzie JE, Bossuyt PM, Boutron I, Hoffmann TC, Mulrow CD, et al.. The PRISMA 2020 statement: an updated guideline for reporting systematic reviews. Bmj. 2021 Mar 29; 372.
